# Gender Differences in Sustained Attentional Control Relate to Gender Inequality across Countries

**DOI:** 10.1371/journal.pone.0165100

**Published:** 2016-11-01

**Authors:** Elizabeth Riley, Hidefusa Okabe, Laura Germine, Jeremy Wilmer, Michael Esterman, Joseph DeGutis

**Affiliations:** 1 Geriatric Research Education and Clinical Center (GRECC), VA Boston Healthcare System, Boston, Massachusetts, United States of America; 2 Harvard Medical School, Cambridge, Massachusetts, United States of America; 3 Center for Human Genetic Research, Massachusetts General Hospital, Boston, Massachusetts, United States of America; 4 Department of Psychology, Wellesley College, Wellesley, Massachusetts, United States of America; 5 Department of Psychiatry, Boston University School of Medicine, Boston, Massachusetts, United States of America; Philipps-Universitat Marburg, GERMANY

## Abstract

Sustained attentional control is critical for everyday tasks and success in school and employment. Understanding gender differences in sustained attentional control, and their potential sources, is an important goal of psychology and neuroscience and of great relevance to society. We used a large web-based sample (n = 21,484, from testmybrain.org) to examine gender differences in sustained attentional control. Our sample included participants from 41 countries, allowing us to examine how gender differences in each country relate to national indices of gender equality. We found significant gender differences in certain aspects of sustained attentional control. Using indices of gender equality, we found that overall sustained attentional control performance was lower in countries with less equality and that there were greater gender differences in performance in countries with less equality. These findings suggest that creating sociocultural conditions which value women and men equally can improve a component of sustained attention and reduce gender disparities in cognition.

## Introduction

Gender differences in cognition have been a source of curiosity and conflict for decades. Most known gender differences have small effect sizes, though some isolated examples such as mental rotation are in the moderate range [1]. Some differences (e.g., math ability, [2])have diminished significantly over the last 30 years, presumably due to the changes in social constructs that were driving the inequality [1]. Furthermore, some studies show differences present in majority ethnic groups yet absent in minority ethnic groups. For example, among Caucasian American teenagers, more boys than girls score in the 99^th^ percentile of mathematics achievement, but the opposite is true of Asian Americans [3]. Gender differences may be absent in privileged social classes (e.g., male advantage in vocabulary in lower-caste but not upper-caste Indian children, [4]) or smaller in countries with greater gender equality [5]. These changes over time and differences between ethnic/sociocultural groups challenge the notion that all gender differences in cognition are innate, and increase the likelihood that many are driven, at least in part, by social variables. The current study examined gender differences in sustained attentional control across a large sample, and then correlated these differences with sociocultural conditions across countries in order to better understand the potential sources of the differences.

Sustained attentional control is the ability to maintain selective attention to a task for a prolonged period of time while resisting internal and external distraction. Sustained attentional control is important to daily functioning and has been associated with lapsesof attention in everyday life [6], academic performance [7], and driving ability [8]. Sustained attentional control is often operationalized by Not-X continuous performance tasks [9], which require responding the majority of non-target stimuli while inhibiting responses to rare, temporally unpredictable targets (e.g., Conners, SART, gradCPT). Good performance on these tasks not only involve vigilance but also draws on executive functions including response inhibition and distractor suppression [10]. Sustained attentional control is heritable [11] but can also be influenced during development by factors like childhood adversity and stress [12,13], or enrichments like exercise, computer games, and certain forms of education [14,15]. Gender differences in SAC could be produced or widened by unequal application of the aforementioned factors.

Gender differences in sustained attentional control are incompletely characterized. Some studies show no effect of gender on sustained attention [16], while others suggest that men may have greater vigilance [17], and women may have enhanced inhibitory control [18]. Consistent with a female advantage in inhibitory control, on a continuous performance task, women have been shown to be less impulsive, slower and more variable than men [19]. However, we are not aware of any studies of SAC that took into account sociocultural factors, and we are aware of only one such study of executive function [20]. Studying European countries, Weber et al. found a gender difference in an executive function measure (category fluency) and, importantly, that this difference was reduced in countries with improved living conditions and greater gender equality in education. Our study expands on this work by using a new measure, a more diverse, worldwide sample with a larger age range, and an expanded set of sociocultural country indices.

In particular, we examined sustained attention with the gradual onset continuous performance task (gradCPT, [21,22])using large samples from testmybrain.org (N = 21,484) and including a broad range of countries of origin. To determine whether significant gender differences in performance had sociocultural associations, we employed widely used country indices of gender equality and human development.

## Methods

### Participants

Our participants were 21,781 unpaid volunteers between the ages of 10 and 70 years. Participants visited TestMyBrain.org, a cognitive testing website, over 14 months in 2014 and 2015. TestMyBrain.org is a citizen science website which people can visit voluntarily to become participants in a variety of neurocognitive tasks. The majority (68%) of TestMyBrain.org traffic comes from search engines with the top search terms being “brain tests”, “test your brain” and “mind tests”. The remaining traffic comes from a variety of social media and news sites, with less than 1.5% of traffic per site. Data from TestMyBrain.org has been shown to be of comparable high quality when compared with data gathered in a lab setting [22,23]. Participants are provided with individualized feedback following each task. Before starting a task, participants give written informed consent, which is stored electronically, for the study which was approved by the Committee on the Use of Human Subjects at Harvard University and the Wellesley College Institutional Review Board. The consent form is given in English regardless of the participant’s country. Our data set did not include repeat participants. Of the 21,484 remaining participants, there were 11,612 males (age *M* = 31.27 years, *SD* = 12.91) and 9,872 females (*M* = 29.77 years, *SD* = 13.81). For each participant we collected age and gender, whether English was the native language, and ethnicity (all questions in English), but no other biographical information. To identify where the gradCPT was performed, we used location information as gathered from the participant’s IP address.

### Task and procedure

The gradual onset continuous performance task (gradCPT), designed to measure sustained attentional control, was presented at TestMyBrain.org as previously reported by our group [22]. In the task, participants are shown a series of gray-scale images which gradually transition from one to the next every 800 ms. Each image is either a city scene (nontarget stimulus, 90% of images) or a mountain scene (target stimulus, 10% of images). The gradCPT requires participants to respond by pressing a key to frequent city scenes and withhold their response to rare mountain images (go/no-go task). Because the stimuli transition quickly, discriminating cities from mountains and withholding one’s response to a mountain image is challenging. The gradCPT, in contrast to other continuous performance tasks, avoids abrupt stimulus onsets that can exogenously capture attention, while still requiring responses, leading to reliable measures of response time.

Before beginning the task each participant was given 3 30 sec practice sessions. Data was discarded from any participants who had a prolonged period (30 s or more) without a response. 297 additional participants were excluded because their cognitive task performance deviated more than 3 standard deviations from the mean either on reaction time, variability of reaction time, omission errors, or commission errors.

### Analyses

We calculated 4 main dependent variables: reaction time (in milliseconds, as detailed previously in [21,22,24]), the coefficient of variation (CV, i.e., the standard deviation of reaction times divided by the mean reaction time), commission error rate (rate of erroneously responding to a mountain scene) and omission error rate (rate of failing to respond to a city scene). For followup analyses, we also calculated 2 additional variables, *d’* and criterion. For all further analyses we regressed out the effects of age, using age-corrected residuals calculated using our previously published equations [22]. This was necessary to accurately compare performance between genders, since age has a strong effect on all 4 dependent variables and the men and women in our sample had different average ages (*M* = 31.26, *SD* = 0.120 years vs. *M* = 29.7, *SD* = 0.139 years, respectively). Furthermore, we applied a natural log transformation to the CV variable in order to ensure that the absolute value of skewness and kurtosis was less than 0.5 for all variables.

In our initial analysis we sought to test for a gender difference in performance across all variables using a between-groups MANOVA. We followed up this MANOVA with between gender ANOVAs of each dependent variable.

The next set of analyses compared gender differences in task performance across countries (N = 16,606, 40 countries). To characterize differences between countries we used 4 sociocultural indices (SIGI, HDI, female/male ratio of participation in the labor force, and the poverty rate). We fit a series of 16 mixed effects models. Four models (for each of the 4 sociocultural indices) were fitted for each of the 4 dependent variables, for a total of 16 models. The models included a random effect for country and fixed effects for gender, sociocultural index, and the interaction between gender and sociocultural index.

To confirm and support these analyses, we examined the data treating each country as a separate data point. In particular, for each country, we calculated the average of each of the 4 dependent variables for men and women. Then the difference between genders for each variable in each country was correlated to each of the 4 sociocultural indices (Pearson’s correlation coefficient). To ensure the robustness of these associations, we repeated the analysis using ranks (Spearman’s rho). Finally, to determine whether the correlations depended more on men’s or women’s performance, we regressed variation in men’s performance from women’s performance (and vice versa), by country, and correlated the residual values with the same 4 indices.

#### Significance testing

Significance was defined as p < 0.05 in all cases. For analyses with multiple comparisons, the Bonferroni correction was applied.

#### Software

Custom MATLAB scripts were used to correct for age, to parse data by age and country, and for correlation analyses. R was used for mixed model analysis (“lme4” library, version 1.1–12, [25]). To determine the statistical significance of the mixed models, degrees of freedom and associated p values were calculated using the “lmerTest” package (version 2.0–32, [26]). SPSS was used for MANOVA/ANOVA analyses.

## Results

### MANOVA shows differences in sustained attention between men and women

We first sought to determine if there was an overall effect of gender across our four gradCPT dependent variables (CO, CE, RT, CV). Previous work in our group has shown changes in performance of the gradCPT task across the lifespan and since we had differences in age between men and women in our sample, we ran the between-groups MANOVA on age-corrected data (see Methods and [19]). We found that men and women had overall differences in gradCPT performance across all our main dependent measures, which included omission errors, commission errors, reaction time and the coefficient of variance of reaction time, “CV” (F(3, 21,479) = 21.8, p < 0.001, N = 21,484). The overall effect size of gender was small (partial η^2^ = 0.037).

### Gender differences amongst the individual dependent variables

**Fig 1:**
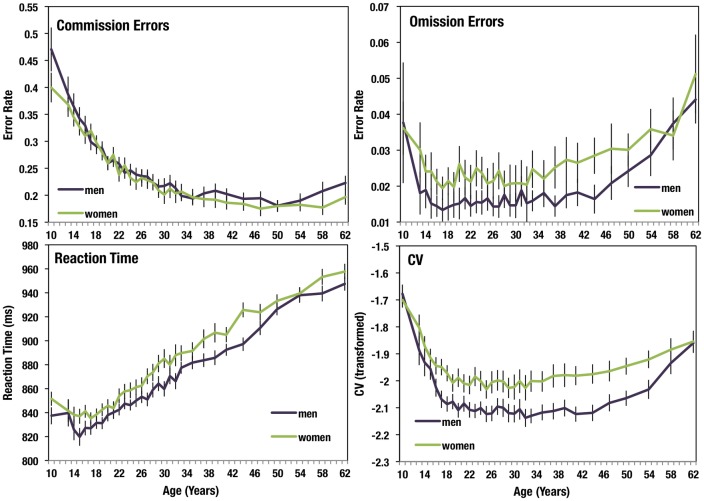
Gender differences in each of the four gradCPT variables across the lifespan. Error bars show 95% confidence interval, N = 11,612 men and 9,872 women, N > 40 in each age bin.

To determine which measures drove the significant overall effect of gender, we separately examined the four variables. Significant gender differences were found individually for all 4 (all p < 0.001, Fig 1, Table 1). Men had faster and more consistent reaction times and made fewer omission errors to non-target stimuli (cities). Women made slightly fewer commission errors than men, but this effect was quite small (Fig 1).

**Table 1:** Effects of gender on each of the four gradCPT variables.

Variable	Mean		Std. Err.		F	p	Partial η^2^ [95% CI]
**RT**	*M*	864	*M*	0.54	F(1, 21,482) = 255	p<0.001	0.012 [0.009 0.015]
	*W*	876	*W*	0.58			
**CV**	*M*	0.13	*M*	0.0005	F(1, 21,482) = 450	p<0.001	0.020 [0.016 0.024]
	*W*	0.14	*W*	0.0004			
**OE**	*M*	0.015	*M*	0.0004	F(1, 21,482) = 146	p<0.001	0.007 [0.005 0.009]
	*W*	0.022	*W*	0.0005			
**CE**	*M*	0.24	*M*	0.001	F(1, 21,482) = 14.4	p<0.001	0.001 [0.0002 0.002]
	*W*	0.23	*W*	0.001			

*Note*. Mean and standard error were calculated by adding the residual values after age correction for men and women, respectively, to the overall group mean. RT = reaction time, CV = coefficient of variation (of reaction time), OE = omission errors, CE = commission errors, M = men, W = women

For completeness, we also examined gender differences in *d’* and criterion (calculated using omission and commission error rates). However, because omission errors and commission errors may have distinct causes, we did not focus on these analyses. On average, women had a slightly lower *d’* (F(1, 21,482) = 102, p < 0.001, partial η^2^ = 0.005) and slightly higher criterion, i.e. more cautious responding (F(1, 21,482) = 266, p < 0.001, partial η^2^ = 0.012).

To determine whether the gender differences in commission and omission error rates were driven solely by a shift in strategy (i.e. making increased omission errors as a result of cautious responding to avoid commission errors), we tested whether there was still a gender difference in omission errors when controlling for commission errors, and vice versa. The gender difference was still significant in both cases (effect of gender on omission errors when controlling for commission errors, F(1, 21,480) = 65.5, p < 0.001, effect of gender on commission errors when controlling for omission errors, F(1, 21,480) = 6.56, p = 0.001).

Besides strategic differences, an additional possibility is that the gender difference in error rates and variability could simply be driven by reaction time differences between genders. However, this was not the case. When reaction time was used as a covariate as well, the gender differences in CV (F(2, 21,480) = 199, p < 0.001), commission errors (F(2, 21,480) = 7.8, p < 0.001,) and omission errors (F(4, 21,480) = 31.5, p < 0.001) all remained significant. Thus the observed gender effects, showing that women make more errors of omission and have a greater degree of fluctuation in performance, while men make more errors of commission, still stand.

### Are the observed gender differences related to sociocultural factors?

Although the effect size of gender in our overall sample was small, we observed that the effect of gender were much larger in certain countries than others within our sample. To determine the source of this variation, we next examined whether or not sociocultural differences across countries were associated with the observed gender differences. If sociocultural factors are significantly associated, it provides evidence against a strictly biological explanation of the gender differences we observed. For our first analysis, we used four valid and reliable indices of sociocultural conditions within a country, the Social Institutions and Gender Index (SIGI, family discriminatory code subscale), published by the Organization for Economic Cooperation and Development Development Center, the Human Development Index (HDI) published by the United Nations Development Programme, the ratio of female-to-male labor force participation, published by the International Labour Organization and the World Bank, and the poverty rate, published by the Central Intelligence Agency (Table 2). These indices were chosen because we hypothesized, based on the results of Weber et al., (2014) that the conditions they represent could affect gradCPT performance.

**Table 2:** Sociocultural country indices examined in the current study.

Index	Description	Source
**SIGI: discriminatory family code**	“This sub-index captures social institutions that limit women’s decision-making power and undervalue their status in the household and the family.”	http://genderindex.org/
**Human Development Index (HD)**	“The Human Development Index (HDI) is a summary measure of average achievement in key dimensions of human development: a long and healthy life, being knowledgeable and have a decent standard of living.”	http://hdr.undp.org/
**Poverty**	“Percentage of population living below the poverty line”	https://www.cia.gov/library/publications/the-world-factbook/
**Women in the labor force**	“Female/male ratio of labor force participation”	http://data.worldbank.org/indicator/SL.TLF.CACT.FM.ZS

We restricted our data set to include only participants whose country location (from IP address) was recorded during testing (N = 16,606, see S1 Table for details). Each of these 16,606 participants was assigned SIGI/HDI/female-male labor force participation/poverty scores based on their country. We used mixed effects models, with a random effect for country and fixed effects for gender, each index, and the interaction between gender and each index. We found that three of our four indices (excluding poverty) were significantly related to gradCPT performance (see Table 3). In particular, less human development and gender equality were associated with slower reaction times, higher CV (more variability), more omission errors and, somewhat paradoxically, slightly fewer commission errors. There was also a significant interaction between gender and three of the four sociocultural indices within omission and commission errors, demonstrating that although overall average performance was affected by sociocultural conditions, men and women were not affected to the same degree (Table 4). There were no significant interactions between index and gender within reaction time and CV, and notably, there was no significant interaction between poverty and gender in any variable.

**Table 3:** Effects of sociocultural conditions on average (men and women together) gradCPT performance.

Dependent Variable	Sociocultural Index	T	P Value
OE	Labor force	T(43) = -7.22	<0.001*
	SIGI	T(51) = 7.26	<0.001*
	HDI	T(42) = -6.79	<0.001*
	Poverty	T(39) = 1.41	0.17
CE	Labor force	T(59) = 3.09	0.003*
	SIGI	T(84) = -3.47	<0.001*
	HDI	T(62) = 2.97	0.004
	Poverty	T(127) = -1.63	0.11
RT	Labor force	T(42) = -3.19	0.003*
	SIGI	T(51) = 3.23	<0.001*
	HDI	T(47) = -3.25	<0.001*
	Poverty	T(44) = 1.16	0.250
CV	Labor force	T(47) = -4.46	<0.001*
	SIGI	T(66) = 4.17	<0.001*
	HDI	T(59) = -6.46	<0.001*
	Poverty	T(47) = 0.875	0.386

*Note*.

* indicates significance after Bonferroni correction (cutoff = 0.003125).

OE = omission error, CE = commission error, Labor force = female/male ratio of labor force participation

**Table 4:** Gender*sociocultural interactions in gradCPT performance.

Variable	Factor	F	FDR-corrected p
OE	Labor force*gender	T(16,420) = 3.87	<0.001*
	SIGI*gender	T(15,610) = -3.73	<0.001*
	HDI*gender	T(16,390) = 3.91	<0.001*
	Poverty*gender	T(14,750) = -1.45	0.146
CE	Labor force*gender	T(16,230) = -4.42	<0.001*
	SIGI*gender	T(14,730) = 4.30	<0.001*
	HDI*gender	T(15,740) = -3.53	<0.001*
	Poverty*gender	T(14,250) = 1.87	0.062
RT	Labor force*gender	T(16,420) = 1.60	0.109
	SIGI*gender	T(15,794) = -1.66	0.096
	HDI*gender	T(16,457) = 1.67	0.095
	Poverty*gender	T(14,733) = -0.078	0.938
CV	Labor force*gender	T(16,420) = -0.626	0.531
	SIGI*gender	T(15,560) = 0.822	0.411
	HDI*gender	T(16,220) = -0.268	0.789
	Poverty*gender	T(14,700) = 0.718	0.473

*Note*.

* indicates significance after Bonferroni correction (cutoff = 0.003125).

OE = omission error, CE = commission error, Labor force = female/male ratio of labor force participation.

To ensure that strategy shifts were not driving these effects, we tested whether there was still a gender*sociocultural index interaction within omission error rate when controlling for commission error rate, and vice versa (i.e. testing for gender*sociocultural index interactions within commission errors while controlling for omission errors). The gender*index interactions remained significant in all cases (S2 Table). Last, to determine if slower RTs were driving the observed significant effects, we subsequently included reaction time as a covariate in all the significant models (commission and omission errors). The overall effects and interactions between gender and sociocultural index all remained significant in all cases.

**Fig 2:**
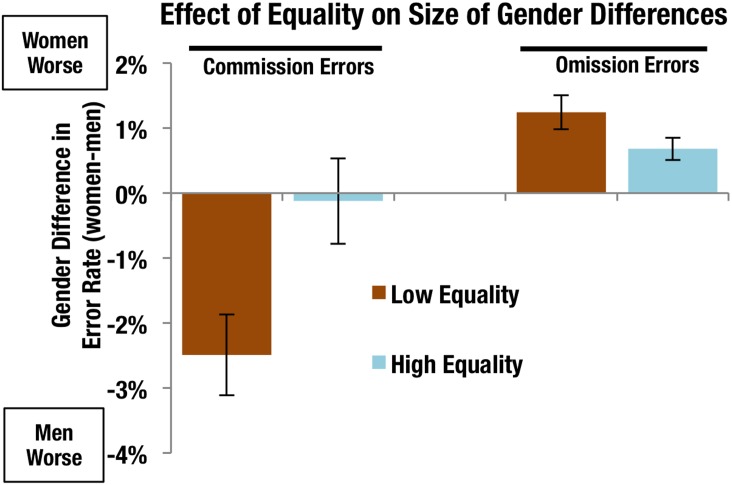
Gender differences in age corrected error rates in low and high gender equality conditions. Error bars show standard error. Low and high equality were defined as the countries in the bottom and top quintile of our sample according to the United Nations’s Gender Inequality Index. N = 8 countries per quintile, low equality N = 2,066 (Egypt, India, Pakistan, Bangladesh, Indonesia, South Africa, Brazil, Phillippines) high equality N = 1,657 (Germany, Sweden, Denmark, Netherlands, Italy, Norway, Belgium, Finland).

For illustrative purposes, the impact of gender inequality on performance can be seen in Fig 2, in which the age-corrected gender differences in error rates are compared between the lowest-equality quintile (lowest 8 countries) and highest-equality quintile (highest 8 countries), according to the Gender Inequality Index (http://hdr.undp.org/en/content/gender-inequality-index-gii). Gender differences were larger in unequal conditions than in equal conditions, with men making more commission errors and women making more omission errors. Gender inequality accounted for a 1–2.5% change in error rates.

#### Correlation analyses using sociocultural indices

**Fig 3:**
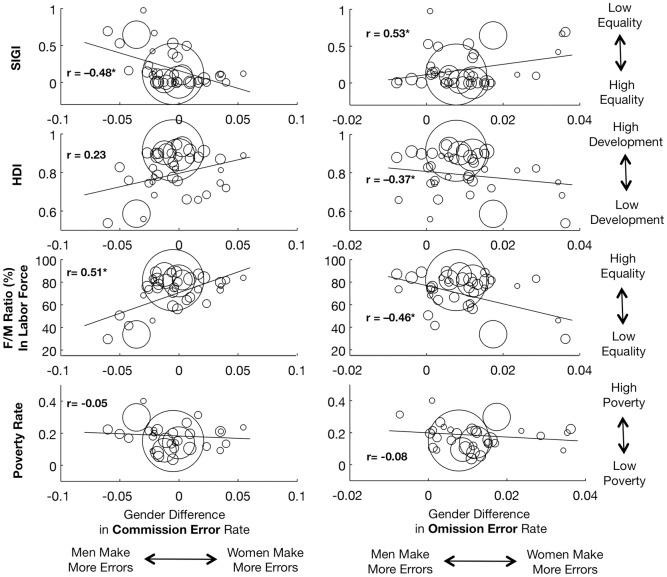
Gender difference in omission and commission error rate versus three sociocultural indices. Social Institutions and Gender Index (SIGI), Human Development Index (HDI), and female/male ratio of labor force participation. Residualized gender difference is average women’s age-corrected score minus average men’s age-corrected score. A negative gender difference indicates that men made more errors than women; a positive gender difference indicates that women made more errors than men. Circle area reflects the number of participants from that country, N = 16,552 people, 40 countries. Linear trendline calculated using unweighted country averages. *indicates significance after FDR correction.

To further confirm that the sociocultural indices were correlated with the size of gender difference, we performed Pearson’s correlation analysis between the average gender difference in each country and each of the 4 indices. For these analyses we excluded a single country from which fewer than 20 women participated (total countries = 40, total N = 16,552). For CV and reaction time we found no significant correlation between the magnitude of gender difference and any indices of social conditions (all |r| < 0.35). However, consistent with the mixed model analysis above, for commission and omission errors we found significant correlations (using the Bonferroni correction) between gender difference and indices of gender equality (Table 5, Fig 3). Specifically, we found that in conditions of lower gender equality, men made more commission errors and women made more omission errors, but as gender equality increased, men and women performed more similarly on both measures. These results, using country averages, supported the results of our mixed model analysis above.

**Table 5:** Pearson correlation coefficients and corresponding p-values for relationships between sociocultural indices and gradCPT gender differences (women—men).

Variable	Index	Pearson’s R	P Value
**CE gender diff.**	SIGI	-0.48*	0.00017*
	Labor force	0.51*	<0.001*
	HDI	0.23	0.16
	Poverty rate	-0.054	0.74
**OE gender diff.**	SIGI	0.53*	<0.001*
	Labor force	-0.46*	0.003*
	HDI	-0.37	0.017
	Poverty rate	-0.078	0.63

*Note*.

* indicates significance after Bonferroni correction (cutoff = 0.00625).

CE = commission error, OE = omission error, SIGI = Social Institutions and Gender Index, Labor force = female/male ratio of labor force participation, HDI = Human Development Index.

#### Isolating variation in men’s and women’s performance

The above analyses of gender differences used difference scores (men minus women), which are the most common way to describe differences in performance between men and women. However, using difference scores implicitly defines a model in which variation in men’s and women’s performances contribute equally and oppositely to the resultant measure. This model does not invariably hold true [27,28]. Therefore, we sought to determine which of two models better characterized the gradCPT gender difference/sociocultural association: 1) when women’s performance is considered the condition of interest and men’s performance was considered the control (i.e. women’s performance drives the effects), or 2) the converse, when men’s performance is considered the condition of interest and women’s performance is considered the control (i.e. men’s performance drives the effects). When we used linear regression to remove variation in men’s scores from variation women’s scores, we found that all of the significant correlations in Table 5 remained statistically significant. However, removing variation in women’s performance from men’s resulted in no significant correlations. Therefore, a model in which women’s performance is considered the control, and men’s performance is the condition of interest, does not fit this data as well as the converse model. This suggests that although there is significant shared variance in men’s and women’s performance, it is the unique variance in women’s performance, not men’s, that varies by gender equality across countries.

#### Effect of sociocultural conditions on *d’* and criterion

When we examined the relationship between *d’*/criterion and sociocultural conditions, we found that criterion scores were significantly correlated to indices of gender equality (r = -0.59 with SIGI, p = 0.0009, r = 0.56 with labor force participation, p = 0.002) and human development (r = 0.51 with HDI, p = 0.006), with women responding more cautiously than men in countries with less equality. Criterion scores were not significantly correlated with poverty rate (r = -0.096, p = 0.632). In contrast, gender differences in *d’* were not related to sociocultural conditions (all |r| < 0.2). These measures, which collapse omission and commission errors into a single number, could obscure the relationships between error type, gender, and sociocultural conditions. Since omission and commission errors might represent different aspects of behavior [27] we decided not to pursue further analysis of d’ and criterion (see Discussion).

#### Additional analyses

The correlations we found were robust to several challenges. We repeated our statistical analysis using ranks (Spearman’s rho) instead of raw data. All of our significant correlations reported in Table 5 remained significant. We also ensured that no significant correlation depended entirely upon either a single country, or upon an outlier index (an index value ± 3 standard deviations from the mean).

## Discussion

### Summary

Examining a large web-based sample, we discovered modest but consistent gender differences in sustained attentional control. In particular, we found that on the gradual-onset continuous performance task (gradCPT), men performed faster and less variably than women, but made slightly more commission errors while women made more omission errors. The size of the difference between men’s and women’s error rates varied across countries and was significantly correlated with indices of gender equality and human development. Notably, variation in women’s performance, not men’s, drove these correlations. Together, our results suggest that worse sociocultural conditions for women are specifically associated with alterations in sustained attentional control.

### Gender Differences in Commission and Omission Errors Are Correlated With indices of Gender Equality

Overall, women made significantly more omission errors than men. Although both men and women made more omission errors in conditions of gender inequality, the size of this gender gap widened as gender equality decreased, such that the largest gender differences in omission error rates were in countries with the least gender equality. Notably, in countries with the greatest gender equality, the gender difference was very small and even virtually non-existent in a few cases (e.g., Finland, Sweden, New Zealand). We also demonstrated that men made significantly more commission errors than women, and that the size of the gender gap in commission errors was also related to gender equality. As conditions became less equal, men made more commission errors, while women made fewer. Our regression analyses showed that it was primarily women’s performance, rather than men’s, that drove the widening gender gap in both commission and omission error rates. This result is consistent with previous work demonstrating that women’s cognition changes more than men’s as the result of societal progress [20], and extends the finding to non-European countries and a wider age range.

The current results are consistent with other go/no-go studies showing that women tend to make more omission errors whereas men tend to make more commission errors (e.g. [29]) and could be explained by either different degrees of attentional failure or strategic differences. Some researchers suggest that omission errors on go/no-go CPTs represent more profound attentional disengagement, while commission errors may represent a less complete loss of attention [27]. This interpretation suggests that women’s tendency to make more omission errors in conditions of low equality could be the result of more serious disengagement. Alternatively, this differential error propensity could be due to general strategic differences between genders (either socialized or innate), with men taking a more impulsive approach and women either being more risk-averse [30] or demonstrating more inhibitory control. The “risk-averse” interpretation is consistent with the analysis of criterion, with women showing consistently more cautious responding than men. However, omission and commission error rates, the overall gender difference, and interactions between gender and sociocultural indices of gender equality remained significant even after controlling for the other error type. Together these results suggest that the effect of gender equality on error rates is not mediated by a strategy shift alone.

It is notable that measures of gender equality predicted the magnitude of gender difference while the poverty rate did not. This suggests that sociocultural conditions that differentially impact men and women may be more relevant to gender differences in cognition than socioeconomic conditions, which may have more of an impact on both genders.

In addition to finding greater gender differences in countries with less gender equality, we also found that overall cognitive performance collapsed across gender decreased in conditions of low gender equality, low education, and low human development (more omission errors, slower reaction time and greater variability of reaction time). This is consistent with previous work showing that cognitive performance can be affected by conditions of low human development, both during development and during adulthood [31,32].

### Gender Differences in Reaction Time and CV Were Not Significantly Correlated with Indices of Gender Equality

Though both reaction time and CV showed significant, moderate-sized gender differences, these differences did not correlate with indices of gender equality. Previous work has similarly shown that men have faster, less variable reaction times than women on continuous performance tasks [19]. These gender differences could be less susceptible to environmental conditions than gender differences in error rates. Though the current results cannot rule out a biological interpretation, a mounting body of evidence suggests that biological sources of gender differences in cognition are less common than previously thought. For example, the gender gap in mathematical ability, once considered to demonstrate a biological difference in cognition, has been closing for the last 20 years and is now considered to be very small or non-existent [3]. Although our results showed that gender differences in reaction time or CV were present across nations and across the lifespan, we cannot rule out the possibility that they are affected by environmental conditions we did not measure or which could not be captured at the nation-wide level.

### Limitations

Though the current findings provide insights into gender differences in sustained attention, they have limitations. Because our participants were anonymous and self-selected, we have fewer data from participants in countries with low gender equality and low human development. In impoverished countries, a variety of issues such as lack of computer access or lack of English fluency may prevent individuals from being recruited into the study, or understanding the instructions. Computer access and English fluency may be less available to women and people of low socioeconomic status, than others. (In low equality countries we had 2 male participants for every female; in high equality countries we had equal numbers.) We suggest that due to these selection effects, we are most likely underestimating the magnitude of the effect of gender inequality, since women living in the most severe conditions of inequality are less likely to have the time, resources, and education to participate in this study. It would be very useful to repeat this experiment with recruitment/advertising and all TestMyBrain.org content translated as appropriate for each country.

### Conclusions

Our results showed significant overall gender differences in sustained attentional control. We found that countries with less gender equality had larger gender differences in sustained attentional control, with women’s performance in countries with the least equality driving this effect. These findings demonstrate the powerful influence that the sociocultural environment can have on fundamental cognitive abilities and adds to growing evidence that many gender differences in cognition are not hardwired. They also provide evidence for the provocative idea that creating sociocultural conditions of gender equality can improve aspects of sustained attention, and reduce gender disparities in cognition.

## Supporting Information

S1 Fig Histograms showing age distributions of participants by country. Horizontal axis—age. Vertical axis—number of participants.
(TIF)

S1 Table Countries included in our sample, and number of participants (men and women) in each country. (DOCX)

S2 Table Gender*sociocultural interactions in commission error rate when controlling for omission error rate, and vice versa. (DOCX)
