# Correction: Gender Differences in Sustained Attentional Control Relate to Gender Inequality across Countries

**DOI:** 10.1371/journal.pone.0170876

**Published:** 2017-01-20

**Authors:** Elizabeth Riley, Hidefusa Okabe, Laura Germine, Jeremy Wilmer, Michael Esterman, Joseph DeGutis

The incorrect index is listed in the caption for Fig 2. Please see the complete, correct Fig 2 caption here.

**Fig 2:**
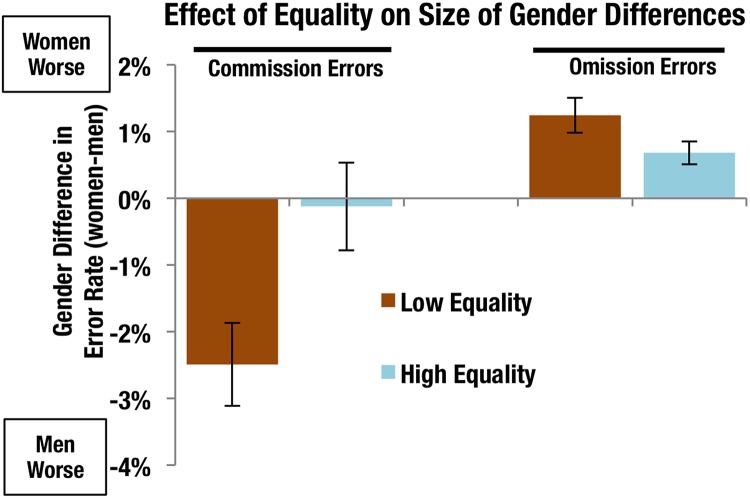
Gender differences in age corrected error rates in low and high gender equality conditions. Error bars show standard error. Low and high equality were defined as the countries in the bottom and top quintile of our sample according to the SIGI. N = 8 countries per quintile, low equality N = 2,115 (Bangladesh, Pakistan, Egypt, India, United Arab Emirates, Malaysia, Philippines, Sri Lanka) high equality N = 3,267 (Denmark, Norway, Netherlands, Germany, Italy, United Kingdom, Ireland, Finland).
