# Supplementary figures and images for: Gender Differences in Sustained Attentional Control Relate to Gender Inequality across Countries
Source: PLoS One. 2016 Nov 1;11(11):e0165100. doi: 10.1371/journal.pone.0165100 (PMC5089545; doi:10.1371/journal.pone.0165100)

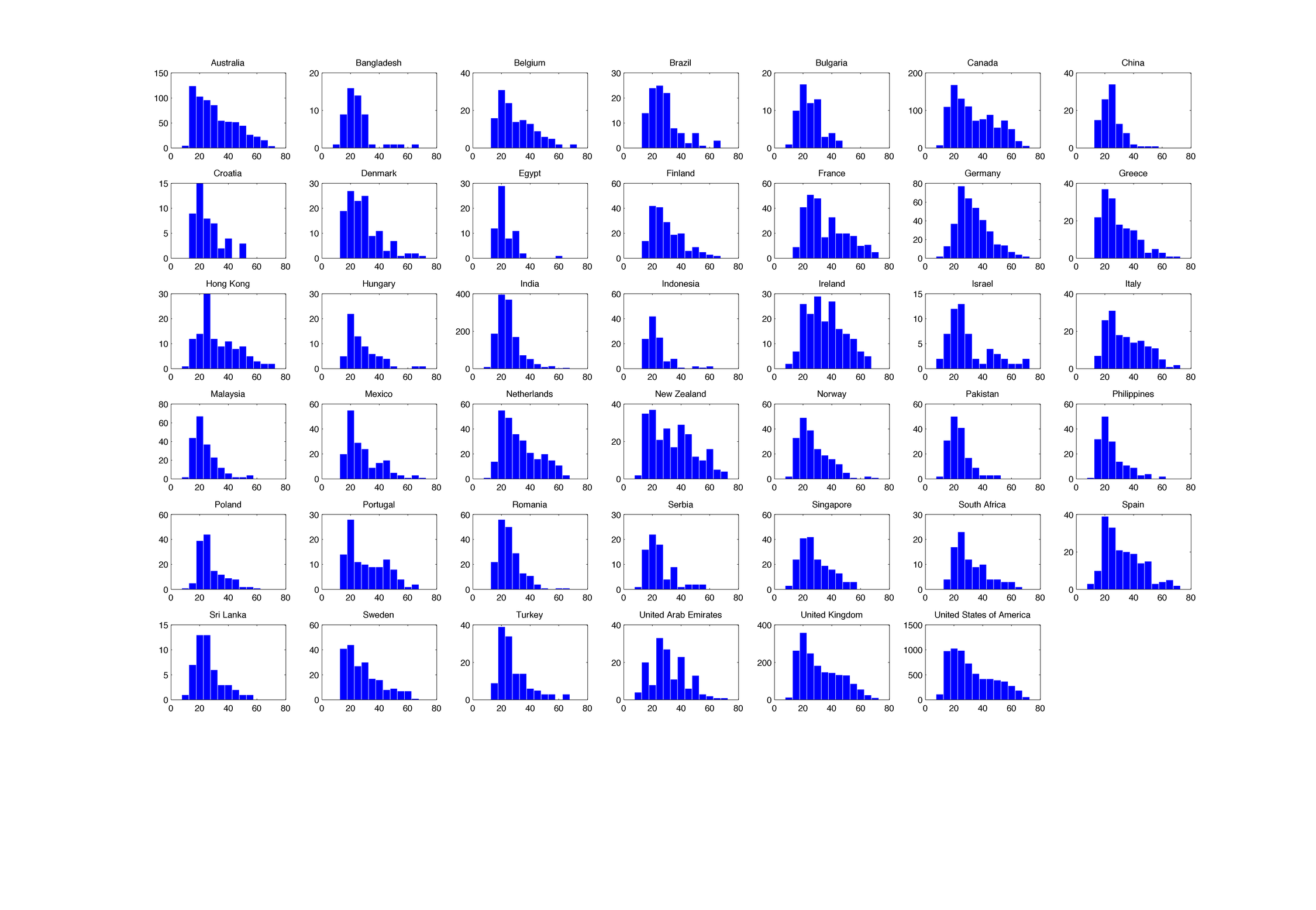

Supplement: S1 Fig — Horizontal axis—age. Vertical axis—number of participants. (TIF) [file pone.0165100.s001.tif]
