# Supplementary material for: Gender Differences in Sustained Attentional Control Relate to Gender Inequality across Countries
Source: PLoS One. 2016 Nov 1;11(11):e0165100. doi: 10.1371/journal.pone.0165100 (PMC5089545; doi:10.1371/journal.pone.0165100)
Supplement: S1 Table — (DOCX) [file pone.0165100.s002.docx]

**S1 Table. Countries included in our sample**

| **Country** | **N Men** | **N Women** |
| --- | --- | --- |
| Australia | 372 | 311 |
| Bangladesh | 45 | 9 |
| Belgium | 78 | 57 |
| Brazil | 66 | 44 |
| Bulgaria | 36 | 26 |
| Canada | 484 | 478 |
| China | 59 | 41 |
| Croatia | 27 | 20 |
| Denmark | 82 | 47 |
| Egypt | 31 | 29 |
| Finland | 104 | 84 |
| France | 148 | 132 |
| Germany | 219 | 137 |
| Greece | 87 | 74 |
| Hong Kong | 79 | 37 |
| Hungary | 37 | 30 |
| India | 894 | 402 |
| Indonesia | 53 | 57 |
| Ireland | 123 | 61 |
| Israel | 37 | 20 |
| Italy | 96 | 63 |
| Malaysia | 87 | 112 |
| Mexico | 112 | 65 |
| Netherlands | 158 | 110 |
| New Zealand | 119 | 117 |
| Norway | 126 | 77 |
| Pakistan | 100 | 52 |
| Philippines | 68 | 87 |
| Poland | 70 | 68 |
| Portugal | 65 | 40 |
| Romania | 95 | 93 |
| Serbia | 36 | 39 |
| Singapore | 120 | 73 |
| South Africa | 50 | 39 |
| Spain | 112 | 68 |
| Sri Lanka | 32 | 17 |
| Sweden | 113 | 92 |
| Turkey | 77 | 51 |
| United Arab Emirates | 95 | 55 |
| United Kingdom | 920 | 860 |
| United States | 2977 | 3416 |
