# Supplementary material for: Gender Differences in Sustained Attentional Control Relate to Gender Inequality across Countries
Source: PLoS One. 2016 Nov 1;11(11):e0165100. doi: 10.1371/journal.pone.0165100 (PMC5089545; doi:10.1371/journal.pone.0165100)
Supplement: S2 Table — (DOCX) [file pone.0165100.s003.docx]

**S2 Table. Gender*sociocultural interactions in commission error rate when controlling for omission error rate, and vice versa.**

| **Dependent Variable (fixed effects)** | **Gender*index interaction** | **T** | **P Value** |
| --- | --- | --- | --- |
| OE (CE, index, gender, gender*index) | Labor force*gender | T(16,420) = 4.04 | <0.001* |
|  | SIGI*gender | T(15,610) = -3.89 | <0.001* |
|  | HDI*gender | T(16,390) = 4.04 | <0.001* |
|  | Poverty*gender | T(14,750) = -1.52 | 0.127 |
| CE (OE, index, gender, gender*index) | Labor force*gender | T(16,200) = -4.56 | <0.001* |
|  | SIGI*gender | T(14,640) = 4.42 | <0.001* |
|  | HDI*gender | T(15,650) = -3.66 | <0.001* |
|  | Poverty*gender | T(14,230) = 1.91 | 0.056 |

*Note.* * indicates significance after Bonferroni correction (cutoff = 0.00625). OE=omission error, CE=commission error, Labor force=female/male ratio of labor force participation, SIGI=Social Institutions and Gender Index, HDI=Human Development Index, FDR= false discovery rate.
